# Voretigene neparvovec for inherited retinal dystrophy due to *RPE65* mutations: a scoping review of eligibility and treatment challenges from clinical trials to real practice

**DOI:** 10.1038/s41433-024-03065-6

**Published:** 2024-04-16

**Authors:** Francesco Testa, Giacomo Bacci, Benedetto Falsini, Giancarlo Iarossi, Paolo Melillo, Dario Pasquale Mucciolo, Vittoria Murro, Anna Paola Salvetti, Andrea Sodi, Giovanni Staurenghi, Francesca Simonelli

**Affiliations:** 1 Eye Clinic, Multidisciplinary Department of Medical, Surgical and Dental Sciences, University of Campania Luigi Vanvitelli, Naples, Italy; 2 Pediatric Ophthalmology Unit, Meyer Children’s Hospital IRCCS, Florence, Italy; 3 Università Cattolica del Sacro Cuore, Rome, Italy; 4 UOC Oftalmologia, Fondazione Policlinico Universitario A. Gemelli IRCCS, Rome, Italy; 5 Department of Ophthalmology, Bambino Gesù IRCCS Children’s Hospital, Rome, Italy; 6 Ophthalmology Unit, S. Jacopo Hospital, Pistoia, Italy; 7 Department of Neuroscience, Psychology, Drug Research and Child Health, University of Florence, Florence, Italy; 8 Eye Clinic, Careggi Teaching Hospital, Florence, Italy; 9 Eye Clinic, Department of Biomedical and Clinical Science, Luigi Sacco Hospital, University of Milan, Milan, Italy

**Keywords:** Hereditary eye disease, Paediatrics

## Abstract

Biallelic mutations in the *RPE65* gene affect nearly 8% of Leber Congenital Amaurosis and 2% of Retinitis Pigmentosa cases. Voretigene neparvovec (VN) is the first gene therapy approach approved for their treatment. To date, real life experience has demonstrated functional improvements following VN treatment, which are consistent with the clinical trials outcomes. However, there is currently no consensus on the characteristics for eligibility for VN treatment. We reviewed relevant literature to explore whether recommendations on patient eligibility can be extrapolated following VN marketing. We screened 166 papers through six research questions, following scoping reviews methodology, to investigate: (1) the clinical and genetic features considered in VN treatment eligibility; (2) the psychophysical tests and imaging modalities used in the pre-treatment and follow-up; (3) the potential correlations between visual function and retinal structure that can be used to define treatment impact on disease progression; (4) retinal degeneration; (5) the most advanced testing modalities; and (6) the impact of surgical procedure on treatment outcomes. Current gaps concerning patients’ eligibility in clinical settings, such as pre-treatment characteristics and outcomes are not consistently reported across the studies. No upper limit of retinal degeneration can be defined as the univocal factor in patient eligibility, although evidence suggested that the potential for function rescue is related to the preservation of photoreceptors before treatment. In general, paediatric patients retain more viable cells, present a less severe disease stage and show the highest potential for improvements, making them the most suitable candidates for treatment.

## Abstract (zh)

*RPE65*基因的双等位基因突变导致近8%的先天性黑蒙症和2%的视网膜色素变性。Voretigene neparvovec (VN) 是第一个批准用于以上两种疾病基因治疗的药物。迄今为止, 实践经验表明, VN治疗后患者的功能有所改善, 这与临床试验结果一致。然而, 目前还没有就VN治疗的患者适应症的特点达成共识。

我们查阅了相关文献, 以回答 VN上市后是否可以提出患者适应症的建议。遵循目标性综述的审查方法, 并通过六个研究问题筛选了166篇论文: (1) VN治疗适应症中考虑的临床和遗传特征； (2) 治疗前和随访中使用的心理物理学检测以及影像学分析； (3) 视觉功能和视网膜结构之间潜在的相关性, 可用于定义治疗对疾病进展的影响； (4) 视网膜变性； (5) 最先进的临床检查方法；以及 (6) 外科手术对治疗结果的影响。

目前关于临床中的适应症的差别, 如治疗前的特征以及之后的结果, 并没有在所有研究中得到统一结论。视网膜变性不能定义为患者适应症的唯一因素, 尽管有证据表明, 挽救视功能的潜力与治疗前光感受器的保护有关。一般来说, 儿科患者保留了更多的活细胞, 病情较轻, 表现出最大的改善潜力, 成为最适合治疗的候选者。

## Introduction

Inherited retinal dystrophies (IRD) are a group of rare genetic disorders that lead to blindness in the majority of affected individuals during childhood or adult life. Leber Congenital Amaurosis (LCA) is the most common form of IRD in the paediatric population. Biallelic mutations in the *RPE65* gene are among the genetic causes of LCA [1, 2]. Despite being an ultra-rare condition, with an estimated prevalence of 1:300,000 affected individuals [3], the spectrum of phenotypes correlated to biallelic *RPE65* mutation is variable and associated, not only with LCA, but also with less severe diseases such as early onset severe retinal dystrophy (EOSRD), severe early childhood onset retinal dystrophy (SECORD), early onset retinitis pigmentosa (EORD) and, in very rare cases, congenital stationary night blindness (CSNB) [4–7]. Commonly associated clinical findings are night blindness, progressive loss of visual field (VF) and central vision, nystagmus, sluggish pupillary reflexes or amaurotic pupils, severely diminished or absent fundus autofluorescence (FAF) and reduced or non-detectable electroretinogram (ERG) irrespective of age of onset [4, 8–11].

Voretigene neparvovec (VN) is the first ocular gene therapy approach approved by FDA in 2017 and EMA in 2018 [12, 13] for the treatment of adult and paediatric patients affected by biallelic mutations in *RPE65*, with sufficient residual viable retinal cells. VN is a viral vector delivering a working copy of the human *RPE65* cDNA to the retina. Upon delivery through a vitrectomy and subretinal injection, vital retinal pigment epithelium (RPE) cells start to produce a functional copy of the *RPE65* gene restoring the visual cycle [14].

Improvement in retinal sensitivity and positive impacts on daily activities have been reported after VN treatment both in clinical trials and real-life [7, 15–17], but there is still a lack of consensus on treatment eligibility.

Indeed, a clinical and genetic diagnosis is insufficient for VN eligibility: a well-structured care pathway to identify who can benefit more from the therapy is necessary, and cell viability evaluation must be included as it is fundamental for treatment response [18–20]. At the moment of VN approval, an expert review group suggested that no specific indication of cell viability should be included in the Summary of Product Characteristics (SmPC), leaving the decision to clinicians [21]. Therefore, cell viability definition remains relatively arbitrary, despite the fact that in a VN clinical trial it has been obtained based on a retinal thickness of more than 100 µm at the posterior pole, on the presence of limited atrophic changes at fundus, or on the remaining visual field [15]. The lack of standardised parameters to define patient eligibility and follow-up steps further complicates the clinical decision, especially in light of the variability of phenotypes, age of onset and disease severity encountered in real life compared to clinical trials [16, 22]. Additionally, treatment decision should consider ethical and pharmaco-economics implications within different National Health Systems.

A clear indication of the sufficient number of vital cells and a characterisation of the limits of retinal and/or functional degeneration (beyond which treatment is not beneficial) would be of great value in clinical practice for defining patient eligibility. To this purpose, a group of Italian experts reviewed the existent literature to investigate whether the therapeutic efficacy can be explained in light of the existing results. Furthermore, the experts explored whether a preferential treatment window could be defined based on the age and clinical features of the treated patients. The final aim is to understand whether limits of functional and/or retinal structure degeneration are already described and to provide guidance for VN treatment eligibility.

## Methods

### Expert panel and study design

A panel of eleven Italian experts (ten ophthalmologists, one biomedical engineer and data manager) with expertise in VN treatment and IRD patient management (paediatric and adult patients) participated in a board to discuss the current evidence regarding the real-world use of VN. The discussion emphasised the need to define patient eligibility criteria for the treatment. The experts analysed the available literature through research questions and collected the information in a scoping review.

### Definition of the research questions

The PRISMA Extension for Scoping Review checklist (supplemental material) was followed to collect, organise and summarise the information [23–25]. The research questions were chosen to gather information on the parameters used to define patient eligibility and investigate whether sufficient evidence is already available to define limits of functional and/or retinal structure degeneration. The scoping review methodology allows to define gaps and needs to be addressed. The research questions were defined as follows:

1. What clinical and genetic features have been considered for VN treatment eligibility?
2. What are the psychophysical tests and imaging modalities used in the pre-treatment and follow-up visits according to age groups?
3. What are the potential correlations between visual function and morpho-anatomic parameters that can be possibly used as biomarkers of disease staging and treatment impact on disease progression?
4. Which parameters are used to define retinal degeneration?
5. Which are the newest/advanced functional and/or testing modalities that will allow us to determine treatment outcomes?
6. What is the impact of surgical procedures on treatment outcomes?

### Search strategy

Articles published in English indexed in PubMed or Embase were searched through specific queries defined by the experts (Supplemental material). Literature search was updated to January 31, 2023. Additional papers were included based on the experts’ opinion (all experts agreed on their inclusion). A first round of selection was made based on the title and abstract by three independent reviewers. A second round evaluated the full text to extract the information related to research questions for the data charting (supplemental material).

### Data organisation and summary

Following the analysis of the data charting, the experts defined ‘relevant’ the clinical studies, post-hoc analyses and data from the real-world setting. All authors discussed the data and organised their comments according to the following areas of interest: A) evaluation of treatment efficacy according to baseline features (clinical phenotype, age, genotype, psychophysical tests and surgical strategy); B) structure-function relationship as a potential determinant of treatment outcome; C) retinal atrophy development after treatment.

## Results

### Characteristics of selected studies

**Fig. 1:**
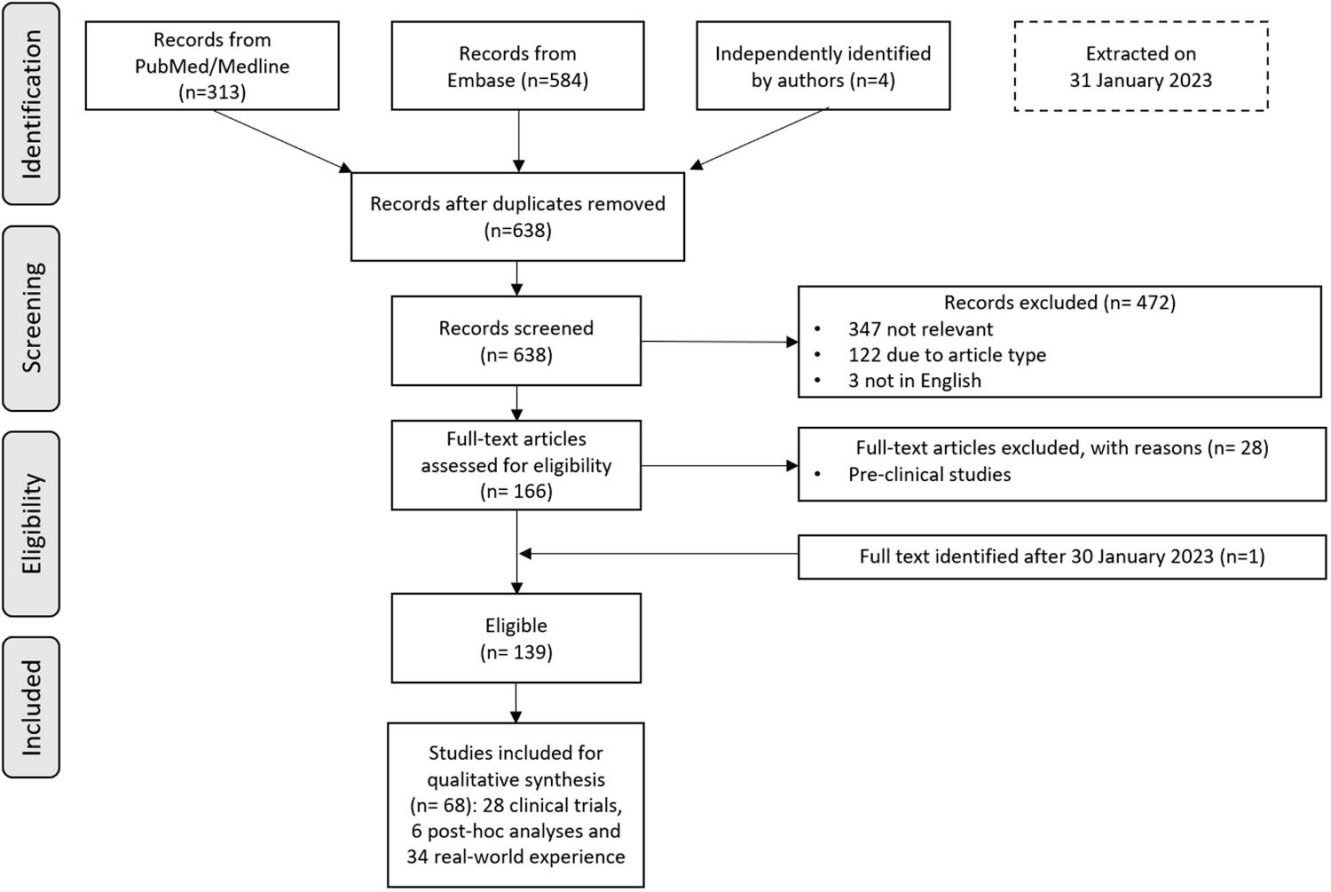
PRISMA flow diagram of the paper selection process. Graphical summary of the paper screening process, with the number of articles found by the different databases and the selection process. Numbers of articles are recorded at the different stages.

One-hundred sixty-six papers were eligible for full text analysis and 68 were considered relevant as they provided original, non-redundant information on VN treatment. Among the relevant papers, 28 were clinical trials, 6 were post-hoc analyses and 34 described real-world experience (Fig. 1).

### Findings of cross-sectional and longitudinal natural history studies: patients’ age, clinical characteristics and disease severity

*RPE65*-associated IRD phenotypes are described in the literature with different terms, as shown by the data charting (Supplemental material).

Visual function degeneration is assessed by best-corrected visual acuity (BCVA), kinetic VF, dark-adapted perimetry and, more recently, full filed sensitivity threshold (FST). The pupillary light reflex assessment was an endpoint only in the phase 1 trial [26]. Recently the evaluation of chromatic pupil campimetry (CPC) has been introduced (see below) [27].

Overall, BCVA is severely compromised since childhood and worsens with age [8]. Consistent findings were provided by different groups [8–10]. A broad inter-individual visual acuity (VA) variability is observed in the first decades of life, and tends to stabilise in early adulthood with progression toward blindness [9], as highlighted by Testa et al., through a time-to-event analysis, predicting a median age of blindness within the fourth decade of life [10].

Similarly, Goldmann VF worsens with age [8, 9, 28], while no clear relationship between age and residual rod function is reported for FST [28]. Although age appears associated with worsening of functional parameters, its role as an absolute criterion for patient eligibility is not confirmed yet. Residual rod function is likely related to preserved photoreceptors rather than age.

We analysed phenotypes and disease severity based on eye fundus, outer nuclear layer (ONL) thickness and age in cross-sectional and longitudinal studies to gather a more comprehensive overview of the patient population and identify the stage that may preclude the treatment.

Fundus abnormalities worsen from the second decade of life, as reported in cohorts of either paediatric or adult patients with LCA or EOSRD (age range 1–54 years), and are more pronounced in older patients [8, 29]. However, in the assessment of age-related fundus abnormalities, ethnicity should be considered. In a Chinese cohort of 30 LCA or EOSRD patients aged 1–45 years, age-dependent phenotypes were slightly different from Caucasian patients: maculopathy and bone spicules commonly reported in Caucasian patients at any age were, instead, described only in adulthood in the Chinese cohort [30].

A longitudinal observation of 43 LCA or EORD patients over 5 years showed no changes in fundus appearance; patients with retinitis pigmentosa (RP) fundus appearance were significantly older [10]. An assessment of disease severity by VF and retinal function may give more sensitive information to characterise patients’ eligibility for VN treatment.

Evaluation of ONL thickness may also be useful. However, ONL thickness mapping revealed variability [31], as foveal ONL thickness in a group of 11 patients aged 11–53 years showed normal thickness in ~50% of subjects, despite an abnormally reduced vision. ONL thickness was greater than expected for the level of dysfunction. In a group of 9 paediatric *RPE65*-LCA patients, ONL thickness topography showed that superior-temporal regions appeared to be the least affected by the degeneration, likely due to the higher rod density, making this area a preferential target for vector delivery. Nevertheless, a broad inter-individual difference was reported. To assess disease severity, the authors recommended a detailed cross-sectional retinal imaging regardless of patient age [31].

Further analysis of ONL thickness was performed in natural history studies over a mean of 1.9 years and 3.9 years by Chung and Testa, respectively [8, 10]. Again, age was not associated with disease severity when ONL thickness by optical coherence tomography (OCT) B-scans were considered in 32 out of 70 patients of 6–38 years of age [8]. This finding is consistent with a non-significant decline in central foveal thickness (CFT) over almost 4 years of follow-up in 25 Italian patients. Furthermore, ONL and CFT thinning were reported in the majority of patients, as well as a more frequent alteration of the ellipsoid zone in extrafoveal areas with a minority of eyes having signs of RPE atrophy [10].

In another longitudinal study [9], 12 patients aged 5–19 years and 8 adults showed a mean reduction of retinal thickness and of ONL and outer retinal layers thickness, while the ganglion cell layer was preserved. Retinal architecture resulted well preserved in the youngest patient.

Dark-adapted static perimetry was evaluated in 17 patients to provide a disease staging: the visual deterioration occurs in sequence, starting with uniform vision loss in the periphery, followed by a more prominent loss in the mid-peripheral area eventually culminating in a complete scotoma within the 30° to 60° range, which expands to involve the central field of vision [28].

As emerged from natural history and cross-sectional studies, *RPE65*-LCA or EOSRD patients deal with progressive functional and structural deterioration since birth or infancy but in the meantime, disease progression rate and severity might widely differ regardless of age. This should be considered when assessing eligibility for treatment: multiple functional and structural parameters are needed to characterise each patient and to determine the potential for functional rescue. Disease severity staging and progression have been analysed in patients within their fifth decade, but elder patients might be encountered in real life.

### Psychophysical tests and imaging performed pre-and post-treatment for *RPE65* patients’ evaluation

Eighty-nine patients, ranging from 4 to 44 years, diagnosed with LCA or EOSRD caused by *RPE65* biallelic mutation were included in clinical trials investigating the safety and efficacy of 3 types of vectors [15, 26, 32–34]. In these studies, functional and structural disease severity overlapped. To avoid bias when interpreting real-world treatment results, we analyse only VN clinical development, which included 41 patients with 81 eyes: Table 1 lists inclusion criteria for VN clinical development.

**Table 1:** Main inclusion criteria for VN phase1/2 and 3 clinical trials [7, 15].

Clinical feature	Inclusion criteria
Age	≥8 years in phase 1/2, ≥3 years in phase 3
VA	Patient should show one of the following: • ≤20/160 in the eye candidate to treatment • worse than 20/60 for both eyes
Viable cells^a^	Defined as either: • an area of retina within the posterior pole of >100 μm thickness measured by OCT scan • ≥3-disc areas within the posterior pole of retina without atrophy or pigmentary degeneration • a remaining VF within 30° of fixation as measured by a III4e isopter or equivalent meridian as measured by a III4e isopter or equivalent (both eyes)

*VA* visual acuity, *VF* visual field.

^a^Evaluation of sufficient viable cells was performed by non-invasive tests, such as optical coherence tomography (OCT) and/or ophthalmoscopy and/ or visual field.

Clinical trials demonstrated the beneficial effect of VN treatment [7, 15]. One of the major challenges in the clinical development of a therapy for IRD patients with severe visual impairment is the definition of the therapeutic efficacy. Functional vision improvement, measured by a validated multi-luminance mobility test (MLMT), was the primary endpoint in the clinical trials [15]. Patients in phase 1 and 3 trials presented a similar MLMT improvement [15, 26, 35]. Moreover, the improved score at day 30 remained stable during follow-up (4 years) [36]. FST was also used as an endpoint assessment and was improved after one year and stable up to 4 years after treatment [36]. MLMT is not routinely used in the majority of clinics, while FST change over time can be considered a marker of functional vision improvement [37].

Noteworthy, the analysis of individual subject data reported in the follow-on study [35] showed that out of the 10 subjects who received treatment in both eyes, only two patients (CH06 and CH12) experienced minimal benefit in the second eye. Specifically, for patient CH06, an additional mutation in the RH12 gene was discovered. Patient CH12 was the eldest (46 years old) with the worst visual acuity score (Hand Motion). However, the first two patients treated in the phase 1 study had Hand Motion visual acuity in their most affected eye and improved visual acuity score, central VF and pupillometric response as described in the initial report of the phase 1 study [7]. The analysis of single patient data from the phase 3 study showed that the only patient for whom the MLMT score did not improve (nor FST, VA, or VF) was the one with the most severe clinical phenotype (unable to undergo MLMT at the highest brightness level before treatment) [15]. These data suggest that the marked reduction in functional parameters is associated with more advanced retinal degeneration and may indicate lower therapeutic benefit. However, from the available information is not possible to evaluate whether this difference in efficacy is related to the different preservation of the retinal structure, as the morphological information provided was limited due to the poor quality of the OCT scans acquired with time-domain technology, and to the presence of nystagmus in several patients [7]. The lack information related to the retinal layers condition does not allow to evaluate treatment impact on disease progression over time.

Following VN marketing authorisation, patients treated in clinical settings are in total 103. They are affected by LCA or EOSRD with ages ranging between 2–44 years. Their clinical features are similar to those described in clinical trials (see Table 1), likely due to the adoption of the same eligibility criteria, despite the different treatment reimbursement policies among countries. In Italy, Health Authority set eligibility criteria as follows: age ≥3 years, VA ≥ 0.5LogMAR and retinal thickness >100 µm. These parameters alone cannot be considered fully reliable biomarkers of a sufficient number of viable cells and cannot distinguish between RPE and photoreceptors’ conditions [4]. Furthermore, VA worsening over time is not considered as a criterion despite in some cases the disease can progress faster than what observed in the natural history study [8].

Table 2 reports the summary of patients’ characteristics and the main results obtained in five real-world studies with at least four treated patients [16, 17, 27, 38, 39]. Patients’ baseline characteristics were not homogeneously reported, which hinders the comparison, but eligibility criteria reflected those used in the VN phase 3 trial: a wide range of BCVA and Goldman VF data were reported in only three studies [16, 17, 38]. In addition, only three studies [16, 17, 38] evaluated light sensitivity threshold with FST in response to white colour stimulus, but only Sengillo et al. described the autofluorescence patterns at baseline [16].

**Table 2:** Summary of real-world data from a retrospective analysis of cohorts comprising at least four patients.

Authors, Year, Ref	Study design	Patient N, age range	Baseline characteristics	Follow up time	Main findings
Stingl et al. [27]	Single centre	5; 14–36 years	Patient’s eligibility to treatment in accordance with the German Society of Ophthalmology recommendations Clinical diagnosis: EOSRD; BCVA (decimal) range FC-0.2; FST blue average (dB): −3.39; FST red average (dB); 0.83; DAC blue average (dB): −2.91; DAC red average (dB): −1.01; CPC rods average (relMCA): 0.31; CPC cones average (relMCA): 4.12	3 months	VA improved or remained stable in all eyes Age as strong prediction factor with respect to blue light FST gain and the average of the DAC stimuli (cyan) and moderate with respect to the increase of the average macular scotopic CPC response Local retinal volume as a moderate predictor with respect to scotopic CPC response The improvement in the rod function (by CPC) depended on patients’ age and remaining retinal thickness
Sengillo et al. [16]	Multicentre	41; 2–44 years	Paediatric eyes mean BCVA 20/150 (20/40-CF); adult eyes mean BCVA 20/260 (20/70-LP); mean CFTs: 210 mm and 176 mm in paediatric and adult patients, respectively Mean white light FST score: 0.6 ± 3.7 dB	Mean 10 months (range 1 week–18.5 months)	3/4 of eyes remained within one line of baseline BCVA. No significant difference in the mean BCVA changes in adult vs paediatric eyes. Baseline VA did not affect postoperative acuity changes BCVA change from baseline in eyes with and without foveal detachment: no significant difference at follow-ups Mild thinning in mean CFT both in paediatric and adult eyes regardless of intraoperative foveal detachment or age FST mean improvement of 21.1 ± 16.6 dB
Deng et al.[17]	Single centre	14; 4–17 years	Mean FST: −2.0 log cd.s/m^2^ Mean CST: 215 μm (range: 192–247) Mean VA: log- MAR 0.98 (range 0.40–1.70; Snellen equivalent, 20/191)	Median 513 days (167–677 days)	Significant improvements in FST, expansion of III4e isopter, BCVA Significant decrease in mean CST Sub-foveal ONL thickness stable over time
Testa et al.[39]	Single centre	6; 7–17	Mean BCVA: 0.70 ± 0.08 logMAR (Snellen equivalent of 20/100) Reduced central foveal retinal thickness, central foveal ONL thickness and mean ONL thickness of the internal ETDRS ring compared to age-matched healthy eyes	6 months	Significant change in VA and increase of ONL thickness in the internal ETDRS ring BCVA change positively associated with ONL thickness increase in the internal ETDRS ring BCVA improved irrespective of intra-operative foveal detachment Central foveal ONL thickness decrease associated with intra-operative foveal detachment. ONL thickness of the internal ETDRS ring change not associated with intra-operative foveal detachment
Gerhardt et al. [38]	Single centre	4; 3–6 years	Clinical diagnosis: LCA BCVA range: 1.3–0.7 logMAR. Nystagmus Lack of 488 nm autofluorescence in all patients Mobility test failed below 40 lux. III/4 isopter in Goldmann VF testing: not disclosed ERGs and 30 Hz flicker ERGs unrecordable FST reliable in one patient (−9.5 dB)	Mean 18.5 months	Statistically and clinically significant VA improvement at month 6 Damped or amplitude reduced nystagmus Mobility test passed at 4 lux Measurable III4e isopter in three patients Post treatment flicker ERGs recordable in two patients Post-treatment FST measured in two patients and remarkably improved by at least 30 dB in each eye at month 6

*BCVA* best corrected visual acuity, *CPC* chromatic pupil campimetry, *CRT* central retinal thickness, *CST* central subfield thickness, *DAC* dark-adapted chromatic perimeter, *ETDRS* Early Treatment of Diabetic Retinopathy Study, *FST* full-field sensitivity threshold, *LogMAR* logarithm of Minimum Angle of Resolution, *ONL* outer nuclear layer, *relMCA* relative maximal constriction amplitude, *VA* visual acuity, *VF* visual field.

Treatment outcomes were also reported based on different parameters: 2 studies [16, 27] reported VA stability and three VA improvement [16, 17]. Two studies [16, 17, 38] showed improvements in Goldmann VF and four in FST light sensitivity [16, 17, 27, 38].

Age dependence of therapy efficacy has been controversial when comparing VN phase 1 results to other vectors: Maguire et al. found a correlation between age and pupillary light reflexes response in the phase 1 trial with VN, and FST improvement was noteworthy in the youngest patients [7]. On the contrary, in 15 patients treated with another *RPE65* vector, age did not influence FST, transient pupillary light reflex and VA [32].

Different relationships between functional parameters and age have been found in real-life: in a group of five patients aged 14–36 years, dark-adapted campimetry (DAC) cyan stimuli correlated strongly with the age of the patients, while the increase of the average macular scotopic CPC response showed poor correlation [27]. On the contrary VN effect on BCVA seems not to be influenced by age or even by pre-treatment value [16]. Most importantly, the FST consistently improved across all studies and VA improved in the paediatric cohorts [16, 17, 27, 38].

Information on retinal morphology by OCT scans was heterogeneous: reduced central retinal thickness before the treatment was described in three studies [10, 16, 17], and reduced total retinal thickness was observed in 70% of patients by Sengillo et al. [16], while Deng et al. reported a decrease in the mean value of total retinal thickness [17]. Testa et al. reported a significant thickening of ONL in the perifoveal area measured by Spectral Domain (SD)-OCT after treatment, supporting ONL thickness as a marker of efficacy and treatment impact on disease progression [39].

Paediatric patients seem to benefit more from treatment despite functional testing being frequently unreliable. Recent works attempted to list all the tests used across IRD centres for the diagnosis and follow-up and tried to find an agreement for children’s assessment without any appreciable indication so far [3, 40].

New imaging techniques could help in overcoming the difficulties in the early-age paediatric population [41]. In 2020, Levi et al. reported the case of a 9-year-old girl displaying autofluorescence along the VN-treated area confirming treatment efficacy [42]. Despite being promising, quantitative autofluorescence and other advanced imaging techniques are still poorly diffused, not affordable for wide clinical pre- and post-operative assessment, and their utility for young children is still to be demonstrated [43]. Gerhardt et al. reported that FST was reliable as a marker of function in the assessment of only two out of four children. However, the authors reported that these two eyes showed a partial recovery on electroretinography (ERG) that was undetectable before treatment [38].

Finally, the increased interest in patient-reported outcomes and the need for functional tests encouraged the assessment of patient perspective [44, 45].

Overall, gene therapy provided positive results for the treated patients, supporting the validity of clinical trials’ inclusion criteria. However, with some exceptions of very young patients, the age range and phenotypes considered do not represent the heterogeneity of all cases and might exclude potential eligible patients.

### Advanced non-routine tests to evaluate treatment outcomes

Besides the measurements of visual function and retinal structure described above, some authors have attempted to carry out alternative tests to assess visual or retinal structure pre- and post-treatment.

Concerning visual function, functional magnetic resonance imaging (fMRI) objectively demonstrated that the visual cortex recovers function in specific areas of the retina with prolonged visual deprivation, when exposed to AAV2-hRPE65v2, confirming that gene augmentation therapy is effective for brain function [46]. Notably, fMRI can assess the visual function improvement durability [47]. In a clinical trial involving ten LCA2 patients unilaterally treated with VN, a non-invasive multimodal neuroimaging protocol evaluated the potential impact of gene therapy on structural transformations in the brain. The treated eyes showed a remyelination of geniculostriate fibre axons and local modifications within the primary visual cortex, underscoring gene therapy’s involvement in inducing structural changes that contribute to the overall enhancement of visual capabilities [48].

Some studies used peculiar imaging and functional tests to describe treatment outcomes: for instance, Stingl et al. reported improvements in CPC and DAC. CPC is an objective measure of retinal area that has undergone sensitivity improvement, while DAC provides differential maps of rods and cones sensitivity [27]. Both methods could enable a precise determination over time of the treatment effects.

OCT B-scan is currently recognised as the standard procedure to determine the state of retinal layer degeneration in *RPE65* patients and of photoreceptor viability [4, 19, 22, 37]. Besides, other imaging modalities might be transferred to the clinical practice, such as Adaptive Optics (AO) imaging, which gives optical access to individual retinal cells and photoreceptors, and allows a prognostic value in terms of direct visualisation of viable cells. Structural outcomes could be used to monitor the patients’ state. However, biallelic mutated *RPE65* patients display low fixation, which is crucial for good AO imaging [22].

Flood-Illumination Adaptive Optics (FIAO) has been used by Sahel et al. to study a cohort of patients with RP. FIAO is deemed to have some technical advantages over confocal AO scanning laser ophthalmoscope systems (i.e., no image distortion, the possibility to correct uneven fundus, larger fields of view, contrast on additional features such as melanin deposition, a more convenient determination of outer segments orientation) [44]. FIAO allowed the achievement of a consensual interpretation of the collected images and the identification of different phenotype-related photoreceptors mosaic patterns. Unfortunately, no information concerning *RPE65* patients has been provided yet, but this non-invasive imaging should be considered in the future to ascertain cell viability and morphological rescue of photoreceptors. An attempt in this direction was made by Kortum et al., who determined short-term morphological rescue of photoreceptors involved in bleb formation in a 15-year-old EOSRD patient [49].

A barely absent short-wavelength autofluorescence (SW-AF) signal is an indicator of dysfunctional RPE cells [50], but the evaluation of autofluorescence post-intervention was not considered a reliable treatment efficacy outcome, although being indicated as a sensitive tool to monitor chorioretinal atrophy development after treatment [51]. Conversely, quantitative SW-AF (qAF) analysis with colour-coded images showed that the visual cycle was established after VN therapy in two patients up to 6 and 8 years after treatment [42, 52]. The qAF images can be superimposed to intraoperative fundus images and corresponding SD-OCT B-scans, to assess the real transduced area, its maintenance over time, and eventual structural changes documenting disease progression: qAF and SD-OCT analysis should be correlated to retinal sensitivity change to determine disease stage variation. Similarly to FAF, principles of qAF acquisition protocol take into account any variation of ocular media conditions, such as lens opacities or cataract, that can vary over time and after treatment, and require surgical intervention [53].

The change in full field stimulus threshold has been positively correlated to MLMT score changes [37], therefore FST is now accepted as a surrogate measure for the improvement of the patient’s skill in navigating environments at dimmer light after VN administration, and the time-consuming MLMT is not considered mandatory in clinical practice [22]. Nonetheless, the need to characterise patient functional vision should not be overlooked and considered a holistic treatment outcome measuring the level of gained independence in mobility and orientation [44]. Currently, devices based on virtual reality allow to test a prototype of orientation and mobility test in virtual space. Aleman et al. provided proof-of-concept data supporting the use of the Virtual Reality-Orientation & Mobility (VR-O&M) test to quantify the impact of gene therapy on functional vision in IRD. In this study, two *RPE65*-LCA patients were evaluated before and within 30 days of VN treatment. Patients’ improvement in retinal sensitivity was measured as dark and light adaptation (5 log changes in FST). Indeed, after treatment, patients were able to navigate VR more accurately and faster even at lower luminance. This technique is reliable for children’s evaluation (>7 years), less time-consuming, and less expensive compared to conventional MLMT [54]. Despite the advantages, the cost/benefit ratio of this technique should be carefully evaluated in its application.

### Genetics

Patient eligibility for VN treatment primarily relies on genotype confirmation of likely pathogenicity or pathogenicity of both *RPE65* variants, as assessed by adopting the American College of Medical Genetics and Genomics (ACMG) recommendation [55]. Furthermore, a comprehensive genotype analysis is of pivotal importance to ascertain that *RPE65* gene variants are solely causing the disease: in the follow-on study report [35] an additional mutation in the *RDH12* gene was deemed to be the cause of poor outcomes in one patient. Indeed, the genotype-phenotype association might have a role in determining disease progression and ultimately customising the treatment window [10, 56]. *RPE65* mutations are generally described in real-life studies [10, 15–17, 26, 27, 32–34, 38]. However, detailed genotype-phenotype association is not always performed. Banin et al. described a founder mutation causing the lack of *RPE65* expression in the Northern African Jewish community and reported retinal sensitivity improvement after treatment [57]. Bainbridge et al. found instead no correlation between patients’ genotype and their response to treatment [33]. The latter situation is also documented by the VN phase 3 trial. Despite the lack of association between treatment outcomes and genotype, it is worth mentioning that hypomorphic mutations, which may be related to mild and late-onset phenotypes, might cause residual RPE65 enzyme activity, as suggested by different studies [28, 58]. Magliyah et al. described a homozygous c.271C>T (p.Arg91Trp) *RPE65* mutation in three siblings with an atypical late presentation which was deemed to have low amounts of 11-cis retinal production. The residual *RPE65* activity allowed some cone and rod function compared to patients with *RPE65* null mutations [59]. Such genetic signatures could make these patients suitable for VN treatment even at older ages.

Conversely, in some natural history studies, genotype was correlated to progressive degeneration. Kumaran et al. significantly correlated two loss of function variants with an age-dependent deterioration of retinal sensitivity in the central 30° [56]. The time-to-event analysis by Testa et al. showed that the presence of two loss of function variants led to BCVA and VF loss over time [10]. Furthermore, a relationship between genotype and BCVA was shown in patients ≤20 years old in the cohort analysed by Shi et al. Indeed, the mean BCVA resulted to be worse in patients carrying two null allele variants (1.15 ± 0.65 logMAR) than in those harbouring two missense variants (0.72 ± 0.43 logMAR) [30].

Classification of variants based on enzymatic residual or null activity might be considered to interpret therapy outcomes and, as a consequence, eligibility. *RPE65* mutation-associated IRDs pose special challenges for genotype/phenotype correlations, as the phenotype resulting from biallelic *RPE65* mutations presents some recurrent features independent of the type of mutation, even though some atypical presentations cannot be excluded. Different combinations of *RPE65* mutations are associated with a severe phenotype, and some missense mutations may result as null. Alternatively, or in addition, variability in disease severity may result from modifier genes impacting *RPE65* associated cell biology/physiology [8]. Indeed, Pierrache et al. [9] reported a high inter-familial and intra-familial variability in visual function in patients with an identical *RPE65* genotype, and no differences in the disease course in subjects with diverse combinations of variants. This evidence differs from what reported by other natural history studies [10, 56].

The reported genotype–phenotype correlation to treatment outcome seems to be conflicting; however, *RPE65* variants can be used to discern baseline RPE65 enzymatic activity and interpret disease severity. Moreover, the genotype–phenotype correlation could confirm consistency of clinical diagnosis and help define a tailored treatment window that takes into account the outer retinal layers condition at time of diagnosis.

### Surgical technique

The on-label procedure is reported on the SmPC: ‘The product is administered as a subretinal injection after vitrectomy in each eye’ [14]. The surgical procedure for vector delivery, consisting of a three-portal pars plana vitrectomy (PPV) followed by subretinal injection, is a key factor influencing the therapeutic success of VN treatment. The risk management plan of VN requires specific training for surgeons aiming to administer gene therapy.

A well-conducted vector delivery is central, as the majority of adverse events reported in VN phase 1 and 3 trials (vitrectomy and induced macular detachment) were related to the surgical procedure [7, 15]. Procedure-related adverse events might have potential consequences on treatment outcomes, such as a retinal tear, retinal disorder, foveal thinning, maculopathy, macular hole, and macular degeneration [37]. Furthermore, endophthalmitis represents a serious adverse event that can compromise the eye, as reported in the VN follow-on study [35]. The currently recommended surgical procedure is nearly identical to the one used in the phase 3 trial, except for macular tamponade with perfluorocarbon liquid that was removed upon VN marketing [4]. The injection site should be located along the superior vascular arcade, at least 2 mm distal from the centre of the fovea [15]. In clinical practice, 3 PPV and subretinal injections might be slightly different than those recommended for the use of dying corticosteroid, subretinal procedures, the number of formed blebs, and the injected volume. Indeed, treatment outcomes rely on both the appropriate surgical technique and the volume injected. The latter may be potentially compromised by inadvertent leakage from the cannula or reflux from the bleb into the vitreous cavity [60, 61]. For such reasons, it is important to understand how VN is delivered into subretinal space and to verify the consistency of the outcomes.

Analysis of real-word experience of eight case reports [20, 49, 62–67] and six retrospective analyses of cohorts including ≥4 patients [16, 17, 38, 39, 51, 68] allowed to collect surgical details.

In the case reports, a total of 11 patients were described, with ages ranging from 22 months to 39 years old (eight paediatric patients). Standard vitrectomy was performed in each case and the use of preservative-free triamcinolone acetonide was disclosed in three patients [20, 67]. Foot-pedal control injection system was used in all cases but one [66], while balanced salt solution (BSS) pre-bleb formation to induce retinal detachment was performed in two eyes in two different reports [64, 69].

Regarding the number of blebs formed to deliver vector genomes, only Jalil et al. did not provide sufficient information [65], whereas in the remaining reports [20, 49, 62–64] the 0.3 ml containing 1.5 × 10^11^ vector genomes were delivered in two blebs as frequently as one bleb. Furthermore, the entire volume was injected and the intraoperative OCT was frequently used.

The time between the first and second eye injection is consistent with VN SmPC, except for one patient who developed an intraoperative subretinal haemorrhage [20].

Consistency between surgical administration and improvement of retinal sensitivity (measured by FST) demonstrated that the procedure in real practice is safe and effective, and that the areas of viable retinal cells have been correctly identified and targeted. However, some adverse events have been reported, such as intraoperative subretinal haemorrhage, subretinal deposits, and choroidal neovascularization after subretinal haemorrhage [20, 62, 64]. Overall, all adverse events were resolved with no impact on the patient’s outcome.

The cohort of the retrospective analyses included 85 patients, mainly children, with mean age ≤40 years. All the studies but Deng et al. [17] used a foot-pedal controlled injection system, and the preservative-free triamcinolone acetonide to ensure complete hyaloid removal at the targeted injection site, while the staining of the internal limiting membrane was frequent. In none of the retrospective studies, BSS was used to prime the retinal detachment.

Multiple blebs for vectors delivering as well as the injection of a reduced volume were evaluated case by case, and time to second eye injection was consistent with VN SmPC with one exception, reporting a time range of 35–216 days [38].

In a case series, despite a risk minimisation procedure consisting of a double fluid-air exchange, vitritis was described in nine out of 23 eyes (6 patients out of 12). In all cases, patients recovered from intraocular inflammation after corticosteroids administration [68]. Interestingly, in this case series, the development of atrophy in four eyes was reported. However, there is not enough information to define a causative link between surgical procedures and the cause for the development of atrophy [68]. Atrophy development and the related issues will be further discussed in the following paragraph. Deng et al. and Gerhardt et al. reported the administration of VN in paediatric cohorts [17, 38]. The surgical delivery and treatment outcomes reported by Deng et al. well mirrored the results obtained in clinical trials [17]. On the contrary, two patients included in the case series of Gerhardt et al. experienced a rhegmatogenous retinal detachment with macular detachment on day 7 after surgery and an intraocular inflammation 1 week after surgery [38]. These events were promptly managed, the patients fully recovered, and the treatment was beneficial.

Central retinal thinning after treatment seemed to be independent of intraoperative foveal detachment [16], while the association of foveal ONL thickness and surgical foveal detachment was followed either by a stable or negative impact [17, 20].

Although reflux occurrence was not explicitly mentioned, it is worth noting that Kessel et al. experienced the spillage of the vector solution into the vitreous cavity. The double fluid-air exchange was intended to avoid inflammation triggered by possible reflux or leakage [68].

Overall, surgical procedures led to functional improvements that could be ascribed to the delivery of vectors to areas with sufficient viable cells.

### Structure–function relationship as a determinant of treatment outcome

Another possible hint in the definition of patient eligibility could come from the evaluation of the retinal structure and function relationship pre- and post-treatment [4, 28]. Retinas of *RPE65* patients are relatively well preserved, at least in the earliest stages, and the severe functional loss does not mirror the retinal structure [31]. Gene augmentation therapy allows the biochemical rescue of photoreceptors and this structure-function relationship can be considered the starting point when assessing patients’ eligibility for VN treatment. Understanding the structure-function relationship might help identify biomarkers of cell viability [31, 70].

Several studies have characterised retinal structure and function in LCA patients with *RPE65* mutation. However, only a few of them assessed and modelled the relationship before and after gene therapy.

An ideal model of pure retinal degeneration assumes that the function is proportional to the number of surviving photoreceptors and outer segment length. Since both of these parameters are proportional to ONL thickness, linear units of sensitivity loss would be expected to be proportional to ONL thickness. As mentioned, this does not apply to *RPE65* patients; however, residual visual function should stem from actually viable photoreceptors. To confirm that vision loss cannot predict retinal degeneration in *RPE65* patients and to test the colocalization of viable photoreceptors and residual function, Jacobson et al. observed the cross-sectional retinal reflectivity profiles obtained with OCT in eight *RPE65* patients and normal subjects. The relationship between nuclear layer thickness and visual function was examined at the locations with the highest cones or rods densities in normal retinas. *RPE65*-mutant retinas showed greater ONL thickness than what predicted based on the amount of visual loss. In some cases, ONL thickness was preserved compared to controls [31].

Colocalized detection of viable photoreceptors and determination of residual function by ONL thickness and dark-adapted sensitivity mapping confirmed that visual sensitivity was present in most of the region with detectable ONL [28]. Thus, ONL thickness should be a mandatory condition for the treatment in adults, given the lack of a straightforward relationship between age and retinal degeneration severity [31, 70]. On the other hand, dark-adapted sensitivity thresholds might be considered a surrogate measure for underlying viable photoreceptors [28].

In a cohort of North African Jewish patients, presenting a founder *RPE65* mutation, the relationship between retinal structure and visual function was compared to the one identified by the theoretical model to provide the retinal locations that could be potentially rescued [57]. However, a threshold identifying the potential for rescue after treatment has not been investigated.

The structure–function relationship in *RPE65*-patients resulted to be variable also within the retina of every patient. Patient eligibility should therefore be assessed by the disproportion between function and structure. Nonetheless, visual function and retinal deterioration might be differently impacted by the treatment and this should be considered when evaluating the improvement over time. The long-term structure–function relationship has been investigated only within the phase 1 clinical trial NCT 00481546 [71], evaluating the efficacy and safety of rAAV2-CBSB-hRPE65 (IND Number, BB-IND 12824). The yearly rate of protracted loss of sensitivity after the peak response was greater than what predicted by photoreceptor degeneration or by the natural history of retinal degeneration [72].

Long-term evaluation of gene therapy efficacy on retinal function and structure might be biased by the type of vector used. Indeed patients included in VN clinical trials seem to maintain the improved retinal sensitivity for up to 7.5 years [73], although no structure-function relationship was reported. Only recent real-world studies explored this relationship before and after the treatment with VN (see Table 2). Stingl et al. tested visual function and retinal structure in seven eyes of five patients with bi-allelic *RPE65* mutations. The clinical examinations included VA testing, dark-adapted FST, DAC with a 30-degree grid, and a 30-degree grid scotopic and photopic CPC.

The pupil response improvement in the scotopic CPC correlated with the baseline local retinal volume. On the contrary, the corresponding local improvement of the dark-adapted sensitivity in DAC (cyan) did not correlate with the retinal thickness. The pre-intervention retinal volume can be a predictor for the improvement of CPC values after the therapy, but not for the DAC values. The authors interpreted the pupil response to scotopic CPC stimuli as a function of the rod number, whereas the DAC stimulus depends on cell sensitivity that may be related to the length of outer segments. Therefore, the change in CPC rod response may represent the number of reactivated rods in the tested location and the most conserved retinal volume might predict treatment response [27].

In the paediatric cohort of Testa et al., quantitative retinal changes were assessed by SD-OCT and related to VA. The authors observed that a higher improvement in BCVA was significantly associated with a higher increase in ONL thickness in the internal Early Treatment Diabetic Retinopathy Study (ETDRS) ring, (*β* = −0.001; *P* = 0.010) indicating a relationship between BCVA gain and change in ONL thickness at 6 months compared to baseline [39].

The change of mean BCVA was deemed to be related to the increase of the ETDRS internal ring ONL thickness which, in turn, seemed to have converted retinal degeneration progression. These data should be confirmed at longer follow-up and in extended cohorts including patients with different disease stages. Such relationship might depend on the early stage of the retinal degeneration. A conclusion on the positive effect on disease progression cannot be ruled out at least in the youngest patients.

Retinal structure response to treatment has been assessed via other OCT parameters, such as CFT. Sengillo et al. found, in paediatric and adult patients, a mild thinning of mean CFT after surgery, associated with a generally stable VA outcome [16]. Notwithstanding, some eyes presented thicker CFT and concomitant VA improvement. Therefore, the post-operative mismatch between foveal thinning and VA stability should be evaluated case by case to assess how structure and function might be related.

In a paediatric cohort of 14 patients, a significant decrease in the mean central subfield thickness (CST) was detected despite the functional improvement measured by kinetic visual field, FST and VA [17]. Despite the variability of VN’s effect on CRT or CST, retinal degeneration appears to progress also in the youngest, although baseline values were similar to those reported in clinical trials.

To date, structure–function relationship has been investigated through topographical assessment of retinal sensitivity and ONL thickness, or retinal volume assessment. However, ONL thickness and reliable visual function measurements determine whether a patient would benefit from the treatment. Indeed, consequence of intra-operative retinal detachment should be carefully monitored as macular disorders have been reported (see above ‘surgical technique’) and might influence post-treatment structure-function relationship.

### Role of atrophy in determining the eligibility for VN treatment

Progressive atrophy areas developed after gene therapy have been reported by several authors in real-life data [39, 51, 52, 68, 74–76] and are currently one of the most discussed complications of VN subretinal injection. No case of atrophy has been reported in clinical trials [15] and up to 5 years of follow-up [36, 73]. Nonetheless, a recent case report described an 11-year-old patient, included in the phase 3 VN trial, presenting evidence of chorioretinal atrophy at the 8-year post-operative visit [52]. As atrophy can potentially diminish treatment efficacy and impact future gene therapies, our analysis focused on examining the available data to identify any potential red flags associated with the treatment, although a comprehensive evaluation of atrophy is not the primary objective of this work.

We here refer to patients identified according to the criteria established by Gange et al. [74]: patients with chorioretinal atrophy should present (i) areas of atrophy not directly related to the touch-down site of the subretinal cannula; (ii) areas of atrophy progressively enlarged over time. Gange et al. reported that progressive chorioretinal atrophy became noticeable between 1 week and 1 year after surgery (mean time of appearance: 4.7 months) in 18 eyes of 10 treated patients. Atrophy appeared both within and outside the area of the subretinal bleb in 55% of treated eyes, within the area of the bleb in 38.9%, and exclusively outside of the bleb area in 5.5% of the eyes [74].

Another report considering 13 eyes of eight patients described areas of decreased autofluorescence already visible 2 weeks post-treatment [51]. After 3 months, all the treated eyes showed new areas of atrophy that progressed beyond the first year. Moreover, in the combined series of 71 eyes (38 patients), the authors observed atrophy development in 20 eyes of 12 patients (28% of treated eyes). The pattern of atrophy growth was similar in the majority of cases with round lesions visible on the fundus and fundus autofluorescence becoming confluent over months after treatment [76].

Giansanti et al. noticed the first areas of atrophy developing at 6 months after treatment [75], whereas Kessel et al. reported atrophic areas associated with previous inflammatory alterations in four eyes (out of nine) at 6 weeks post-treatment [68].

Several hypotheses have been suggested to explain the onset and progression so far.

The increased metabolic activity of RPE cells and photoreceptors following restored visual cycle has been discussed by two groups [51, 74] as a possible cause of vector toxicity. The metabolic effect-based hypothesis is corroborated by the evidence of a greater improvement in the FST results in the atrophy group suggesting that *RPE65* overexpression might drive atrophy development, as highlighted by the German-American cohort of 71 eyes. In this group, atrophy developed irrespectively of patient sex or treatment site and correlated with the initial change of the dark-adapted FST [76]. When plotting the initial FST change against the age at treatment, younger patients (up to young adulthood) seemed those at higher risk of atrophy development [52]. Nonetheless, in the youngest cohort of patients reported by Gerhardt et al. no chorioretinal atrophy development has been observed after two years of follow-up [38].

A second possible explanation of the pathogenesis of atrophy is either inflammation or an immune response to the injected vector. Clinically significant inflammation was found in 11% of eyes by Gange et al. [74] and in 44% of the eyes described by Kessel et al. [68], where inflammation was more often or more severe in the second eye treated (the one receiving immunosuppressants for a shorter time). On the contrary, Reichel et al. did not observe any sign of inflammation, nor an obvious difference in the onset or extent of atrophy in the second eye. Indeed, in this study, two eyes were treated on average 3 months apart, a longer time interval compared to other groups [51]. Despite in non-human primates pre-existing antibodies to AAV have been correlated to intraocular immune response [77], the adaptive immune response was not assessed in clinical practice as it was not detected in clinical trials [7, 15, 35].

Surgical procedure could also be appointed as a possible mechanism causing atrophy development in areas other than at the retinotomy site: the mechanical trauma applied to the outer retina during the temporary detachment causes the loss of photoreceptor outer segments and it likely compromises RPE integrity (as seen with OCT) [78]. Highly degenerated retinal tissue in *RPE65* patients might impair anatomical recovery, so that the surgically induced retinal detachment might trigger atrophy development [78]. Initial injection speed does not seem to be related to atrophic changes [78].

The presence of myopia has been considered related to atrophy development in the Gange et al. study, where nine of ten patients were myopic, with a variation of refractions between −11.50 and +1.75 dioptres, and eight patients experienced chorioretinal atrophy with similar atrophy in the fellow eye [74]. In myopic patients, the presence of very thin choriocapillaris might suggest a higher susceptibility to choriocapillaritis and thus inflammation, although in the German-American cohort, the spherical equivalent refraction was similar between the atrophy and non-atrophy groups [76].

Finally, despite atrophy development appears to be temporally correlated with VN administration [51], the natural history has not been definitively excluded.

Concerning clinical impact, there was a very limited influence of the atrophic changes on visual function in the first months of follow-up. Indeed, despite atrophy, the VA improved or remained stable in 83% of patients [74] likely because the fovea was spared. An average of 3-log unit improvement in FST indicated a successful response to treatment, which was consistent with results of the clinical trials. Additionally, all eyes improved in perimetry with just 23% of patients affected by paracentral scotomas that could be ascribed to atrophy [16, 74].

Similarly, none of the thirteen eyes retrospectively analysed by Reichel et al. perceived a scotoma, which can be difficult to notice given the poor visual function before gene therapy [51]. Both Giansanti et al. and Testa et al. confirmed the absence of a functional impact upon the atrophic changes in the treated patients [39, 75] as well as in the Kolesnikova et al. study where the patient presented functioning visual cycle as showed by central autofluorescence at 6 and 8-year follow-up, supported also by patient’s BCVA stabilisation [52].

## Discussion

We analysed the available information regarding *RPE65* patient characteristics and post-treatment functional and morpho-anatomical results of VN therapy to highlight indicators for treatment eligibility. However, the current data did not allow a definition of the optimal treatment window due to two main reasons: the characteristics of patients treated in real-life closely overlapped with the inclusion criteria of phase 3 trials, and the outcomes were not homogeneously reported across the studies.

One exception to the inclusion criteria of clinical trials pertains to VA > 20/60 [16], which may be influenced by regulatory requirements in different countries. In patients with preserved VA at baseline (i.e., VA ≥ 20/60), VA improvement does not seem to differ among cases [16], suggesting that VA has a minor impact on determining viable retinal cells number. It is reassuring that the experience with VN in clinical practice aligns with the results of trials even with follow-ups ranging from 1 month to 2 years [20, 52, 63, 65–67]. The available evidence demonstrates the effectiveness of VN in paediatric patients and young adults with a wide range of pre-treatment functional characteristics. It is however impossible to identify a functional threshold that precludes a potential benefit of VN treatment, and no specific clinical phenotypes/genotypes have been identified as better responders.

Although initially conflicting, evidence suggests that the potential for functional rescue is related to pre-treatment preservation of photoreceptors rather than an age threshold [16, 28, 31, 59]. In an Italian cohort of *RPE65* patients, only seven out of 43 patients did not meet the minimum retinal thickness required for inclusion, indicating that the treatment window can be extended to patients ≥30 years [10]. Additionally, cellular survival might be influenced by genetic variants that impact the time of visual function loss, regardless of age [10, 56]. On an individual basis, disease severity is age-dependent. Studies of visual function and photoreceptor topography indicated that retinal degeneration and function can be similar both in the first and third decades of life [28, 79]. Regarding the degeneration stage, the number of sufficient viable retinal cells at baseline has not been defined [10, 16, 27, 38]. Treatment impact on retinal disease progression can be tracked through changes in quantitative CRT and ONL thickness, which decreased over time in all but one study, where ONL increased at 6 months after treatment [39]. However, analysing the structure-function relationship, no ONL thickness threshold has been determined as an indicator of treatment efficacy. Therefore, a general upper limit for retinal degeneration cannot be defined.

For the most appropriate treatment decision, regardless of age, relying on available pre-treatment follow-up data may help to determine the rate of functional and retinal disease progression in each patient. Assuming a comparable number of residual photoreceptors upon injection and a similar RPE condition, the chance of improvement is age-independent [32]. However, paediatric patients are more likely to retain a greater number of viable cells and present a less severe disease stage based on the VF and fundus appearance. The greater potential for improvement makes young patients the most suitable candidates for treatment. Paediatric cohorts treated in the clinical setting show significant VA improvement, though positive FST results can be observed at any age [16, 17, 27, 38, 39].

The development and pathogenesis of atrophy regions remain the most debated issue related to the gene therapy. FAF imaging is a sensitive tool for detecting early changes in retinal atrophy [51], and indocyanine green angiography may be useful in the early detection of choriocapillaritis or choroidal inflammation. While surgical delivery has been listed as a possible cause, atrophy development may likely be due to patient susceptibility. Until a better understanding of this complication’s pathophysiology and its clinical impact are achieved, research is mandatory to establish criteria for better pre-operative evaluation of the risk/benefit ratio, and to modulate the overall treatment strategy (such as adjusting the immunosuppressive therapy or modifying the surgical procedure). Currently, no objective red flags indicate any pre-treatment patient characteristics and/or surgical management that would contraindicate VN therapy due to the risk of developing atrophy.

Real-word data are warranted to further expand the experience with VN and provide a source of information that should be constantly updated and enriched for a better characterisation of baseline features and treatment outcomes. For instance, the EU PASS CLTW888A12401 study [80], a global (ex-US countries) non-interventional registry, aims to collect safety outcomes from real-world practice to determine the long-term efficacy of VN treatment in the largest cohort of *RPE65* patients. To facilitate data comparison, ophthalmologists should report homogeneous parameters to describe patient’s characteristics: phenotype description and clinical diagnosis, genotype-phenotype correlations, psychophysical tests such as FST, VA, kinetic and static VF, retinal layers detection through quantitative and qualitative OCT parameters, colour fundus photography, NIR reflectance and/or FAF to assess RPE cells viability. We believe that non-routine assessments should be further investigated before their implementation in the clinical practice. Due to limitations related to the frequently unstable fixation capacity of *RPE65* patients, scarce test reproducibility should be expected especially when performing AO or qAF imaging.

However, methods mapping retinal sensitivity by distinguishing rods and cones readouts should be adopted in the future.

The subretinal injection technique is continuously being enhanced to safely and precisely deliver available or under development gene therapies. Implementing advanced microscope-integrated optical coherence tomography protocols allows for the objective assessment of parameters affecting surgery success and for defining the actual volume of the medication forming the bleb(s). Consequently, the safety and efficacy of VN treatment could improve in the real practice [60, 61, 81].

## Conclusions and future directions

Available evidence on *RPE65* patient characteristics and VN post-treatment functional and morpho-anatomical results do not identify clear indicators for treatment eligibility, therefore an optimal treatment window has not been determined. However, some practical considerations can be derived. The potential for functional rescue is related to the pre-treatment preservation of photoreceptors rather than to a threshold age, therefore detecting the ONL thickness and reliable measure of residual visual function, such as retinal sensitivity, might be sufficient to warrant treatment. Early intervention might be crucial for better treatment outcomes since paediatric patients seem to benefit the most from VN treatment. Standardising the reporting of comprehensive pre-treatment patients’ characteristics, the description of the surgical technique, including the actual volume of vector solution delivered, and treatment outcomes will enhance the reliability and comparability of research findings, leading to more robust conclusions and recommendations.

## Supplementary information

Data Set

Supplementary Material

## Data Availability

All data are available within the text or the supplementary material.
