# Supplementary material for: Voretigene neparvovec for inherited retinal dystrophy due to RPE65 mutations: a scoping review of eligibility and treatment challenges from clinical trials to real practice
Source: Eye (Lond). 2024 Apr 16;38(13):2504–15. doi: 10.1038/s41433-024-03065-6 (PMC11385234; doi:10.1038/s41433-024-03065-6)
Supplement: Supplementary file 2 — Supplementary Material [file 41433_2024_3065_MOESM2_ESM.docx]

**Scoping review protocol**

**Introduction**

Voretigene neparvovec (VN) is the first ocular gene therapy approach approved for the treatment of inherited retinal dystrophies due to biallelic *RPE65* mutations. Patient eligibility to treatment is not clearly defined, complicating the access to therapy.

A panel of eleven Italian experts with expertise in clinical management VN treated patients (pediatric and adult patients) participated in a board to discuss the current evidence regarding the real-world use of VN, focusing on the possible indication for patient eligibility criteria to treatment. The experts analyzed the available literature through research questions and collected the information in a scoping review.

**Aim of the study**

The primary objective of this scoping review is to summarize the evidence gaps on the definition of an upper limit of functional/retinal degeneration beyond which the VN treatment could not provide a clinical improvement.

**Research questions**

To search for evidence that can drive the patient eligibility to VN treatment, the literature will be searched for the following research questions:

1. What clinical and genetic features have been considered for VN treatment eligibility?
2. What are the psychophysical tests and imaging modalities used in the pre-treatment and follow-up visits according to age groups?
3. What are the potential correlations between visual function and morpho-anatomic parameters that can be possibly used as biomarkers of disease staging and treatment impact on disease progression?
4. Which parameters are used to define retinal degeneration?
5. Which are the newest/advanced functional and/or testing modalities that will allow us to determine treatment outcomes?
6. What is the impact of surgical procedures on treatment outcomes?

**Study design**

A scoping review has the final aim of clarifying key concepts in the literature, identifying and analyzing the current knowledge and the possible gaps [1] and resulted the most appropriated methodology to fulfill experts’ aim. The scoping review follows the methodological approach reported in the literature [2,3]. The PRISMA Extension for Scoping Review checklist was used to report the diagram flow followed during search and paper identification [4].

**Search strategy**

Articles published in English in indexed in Pubmed or Embase were searched using the following query: (luxturna OR "voretigene neparvovec" OR "voretigene neparvovec-rzyl" OR

(RPE65 AND ("gene therapy" OR "biallelic RPE65 mutat*" OR "biallelic mutat*")))

**Eligibility criteria**

Three independent reviewers firstly screened title/abstracts for relevance.

Inclusion criteria:

- Specific reference to gene therapy in patients with inherited retinal dystrophy due to RPE65 biallelic mutations
- Full text available in English

Exclusion criteria:

- In-vitro studies
- Non-peer-reviewed articles (e.g., Conference abstracts / posters / conference paper / conference review)
- Outcomes only related to vectors (e.g., immune response to vector, drug delivery)
- Non-clinical outcomes only (e.g., gene frequency, pathways’ studies or health economics analyses)

Full-texts will be then assessed to acquire information related to the 6 research questions.

**Data charting**

An Excel sheet will summarize the relevant information collected from the included studies, organized according to the 6 areas covered by the research questions.

The following fields were recorded:

- PubMed ID
- First(s) author(s)
- Year of publication
- Paper title
- Journal
- DOI
- Type of study
- Number of patients within the study
- Sex of patients (% males)
- Age of the patients
- Follow-up
- Psychophysical tests, imaging employed (primary studies) /discussed (reviews)
- Patients’ clinical characteristics
- Evaluation of the impact of treatment on disease progression
- Potential correlation between visual function and morpho-anatomic parameters changes after treatment
- Definition of the upper limit of retinal degeneration for VN
- Advanced technologies to be potentially deployed in clinical practice
- Genetic characteristics of patients
- Impact of surgical procedure on treatment outcomes

Each cell of the excel file reports the extract from the paper, with reference numbers related to the original paper.

**Data analysis**

After evaluation of the data charting, the experts agreed to present and discuss narratively the information collected from clinical trials, they post-hoc analyses and real-word evidence, highlighting existing knowledge and the current gaps. Those studies were chosen as they were reporting original information regarding clinical practice, with patients feature and treatment outcome.

As this is a scoping review, the risk of bias assessment will not be performed.

**References**

1. Munn Z, Peters MDJ, Stern C, Tufanaru C, McArthur A, Aromataris E. Systematic review or scoping review? Guidance for authors when choosing between a systematic or scoping review approach. BMC Med Res Methodol. 2018 Nov 19;18(1):143. doi: 10.1186/s12874-018-0611-x.

2. Arksey H, O'Malley L. Scoping studies: towards a methodological framework. International Journal of Social Research Methodology. 2005 2005/02/01;8(1):19-32. doi: 10.1080/1364557032000119616.

3. Levac D, Colquhoun H, O'Brien KK. Scoping studies: advancing the methodology. Implement Sci. 2010 Sep 20;5:69. doi: 10.1186/1748-5908-5-69.

4. Tricco AC, Lillie E, Zarin W, O'Brien KK, Colquhoun H, Levac D, et al. PRISMA Extension for Scoping Reviews (PRISMA-ScR): Checklist and Explanation. Ann Intern Med. 2018 Oct 2;169(7):467-473. doi: 10.7326/M18-0850.
